# Immunosequencing applications in cutaneous T-cell lymphoma

**DOI:** 10.3389/fimmu.2023.1300061

**Published:** 2023-12-21

**Authors:** Jenna Mandel, Laura Gleason, Daniel Joffe, Safiyyah Bhatti, Neda Nikbakht

**Affiliations:** Department of Dermatology and Cutaneous Biology, Thomas Jefferson University, Philadelphia, PA, United States

**Keywords:** cutaneous T-cell lymphoma, immunosequencing, mycosis fungoides, T-cell clonality, high-throughput sequencing of the T-cell receptor

## Abstract

Immunosequencing has emerged as a newer clinical test for assessment of T-cell clonality in the blood and skin of cutaneous T-cell lymphoma (CTCL) patients. Utilization of immunosequencing, also known as high-throughput sequencing of the T-cell receptor (HTS-TCR), enables identification and quantification of the precise genetic signature of dominant T-cell clones. Although immunosequencing is more sensitive than commonly used methods such as polymerase chain reaction (PCR) paired with capillary electrophoresis or flow cytometry, it remains underutilized for CTCL management. Nonetheless, incorporation of HTS-TCR in clinical practice offers distinct advantages compared to other molecular analyses that may improve diagnostic evaluation, prognostication, and disease monitoring in CTCL. The objective of this comprehensive review is to provide a thorough explanation of the application of immunosequencing in the context of CTCL. We describe the significance of T-cell clonality and the methods used to detect it, including a detailed comparison between PCR paired with capillary electrophoresis and HTS-TCR. The utilization of immunosequencing in the blood and skin of CTCL patients is discussed in depth, specifically outlining how HTS-TCR can assist in diagnosing CTCL, predicting outcomes, and tracking disease progression. Finally, we address the potential applications of immunosequencing in clinical management and research as well as the novel challenges it presents.

## 1 Introduction

### 1.1 Cutaneous T-cell lymphoma

Cutaneous T-cell lymphoma (CTCL) represents a subset of non-Hodgkin lymphoma characterized by proliferation of malignant T-lymphocytes in the skin. Among CTCL subtypes, mycosis fungoides (MF) is the most prevalent, accounting for over 60% of all CTCL cases (1). MF is a slow-growing T-cell lymphoma, predominantly of CD4 lineage. It primarily resides in the skin, but can progress to involve the blood, lymph nodes, and viscera in advanced stages. Early-stage MF, also known as the patch/plaque stage, is often marked by large pruritic lesions of variable size, shape, and color that appear in non-sun exposed areas. Lesions may persist for several years before progressing (2, 3). Early MF patches and plaques frequently mimic benign inflammatory dermatoses in both clinical and histopathologic features (4, 5). Consequently, early-stage MF has historically been challenging to diagnose, averaging several years to reach a confirmed diagnosis (6). National Comprehensive Cancer Network (NCCN)/International Society for Cutaneous Lymphoma (ISCL) guidelines call for multiple integrated criteria to aid in diagnosis of early MF including clinical, histopathologic, immunophenotypic, and molecular characteristics (5, 7). T-cell clonality tests are now an essential component of the diagnostic toolkit for CTCL, enabling diagnosis and differentiation of mostly monoclonal early-stage MF from primarily polyclonal benign conditions (4). For nearly fifteen years, polymerase chain reaction (PCR) paired with capillary electrophoresis had been the standard for identification of a dominant T-cell clone in MF patients (8). This standard has recently been contested by the development of high-throughput sequencing of the T-cell receptor (HTS-TCR), also known as next generation sequencing (NGS) or immunosequencing, which has emerged as a more targeted approach for detection of malignant T-cell clones. Immunosequencing precisely identifies and quantifies the associated genetic signature of malignant T-cell clones. As it becomes more widely incorporated into clinical workflows, HTS-TCR provides a platform for broadened applications in the areas of diagnosis, prognosis, and disease monitoring. Herein, we present a comprehensive review for the current state of immunosequencing utility in the prospective landscape of CTCL clinical practice.

## 2 T-cell clonality

### 2.1 T-cell clonal diversity

Each T cell possesses its own distinct T-cell receptor (TCR). TCRs are heterodimeric proteins comprised of either alpha (α)/beta (β) or gamma (γ)/delta (δ) subunits, the former being the predominant type (9). The unique identity of a given alpha, beta, gamma, or delta TCR subunit is based on the sequence of a DNA segment called the complementarity determining region 3 (CDR3). Within each expressed TCR, the CDR3 sequence dictates the antigen binding specificity, essentially serving as a fingerprint or genetic signature for T cells. During T-cell development, variable (V), diversity (D), and joining (J) gene segments undergo somatic V(D)J recombination to create the CDR3 sequences of the TCR subunits. In TCRβ and TCRδ loci, VDJ recombination starts with D to J rearrangement followed by V to D-J rearrangement. Conversely, direct V to J rearrangements occur in TCRα and TCRγ loci (10). Random nucleotide deletions and insertions occur between V(D)J segments resulting in unique combinations that contribute to the diversity of CDR3 sequences. V(D)J recombination generally occurs in sequential order in the nucleus beginning with TCRδ rearrangements, followed by TCRγ, TCRβ, and TCRα respectively. As a result, a given T cell may have up to two rearranged CDR3 alleles of TCR delta, gamma, beta, and alpha in the nucleus while only two sequences are selected to be expressed at the protein level. In alpha/beta T cells, once the first allele of TCRβ begins to rearrange, the rearrangement in the second TCRβ allele may not proceed if a productive amino acid and a resulting functional protein is produced by the first rearrangement. In addition, once the cell proceeds to have TCRα rearrangements, the previously rearranged TCRδ sequences will be deleted (10).

**Figure 1:**
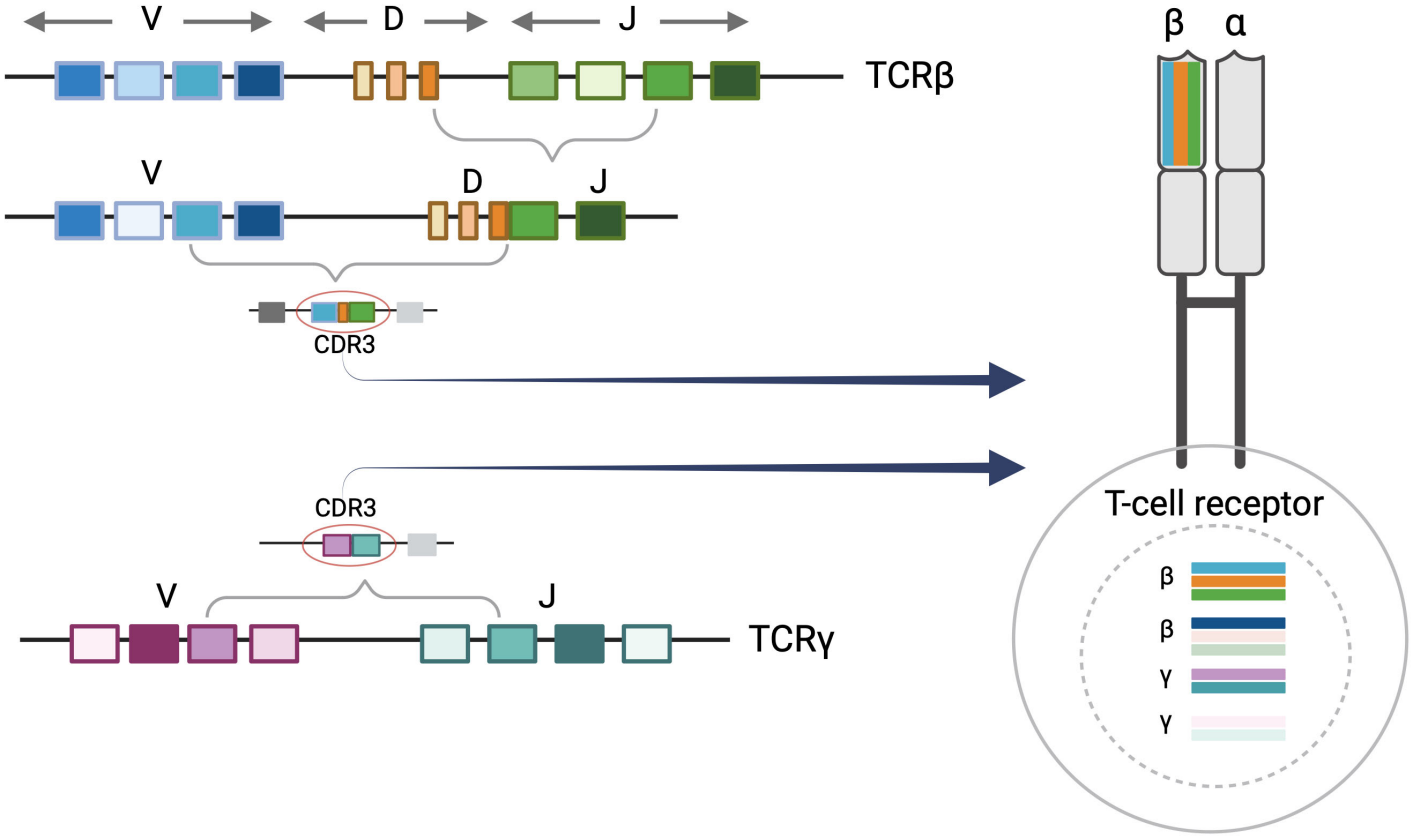
T-Cell receptor rearrangements. T-cell rearrangement is depicted for alpha/beta T cells. Each T-cell receptor (TCR) is comprised of two protein subunits (either one alpha and one beta chain or one gamma and one delta chain). Every TCR has a unique complementary determining region 3 (CDR3) sequence which encodes a distinct major histocompatibility complex (MHC) peptide binding region of the T-cell receptor. This is generated by the V(D)J recombination event during T-cell development. There can be up to two beta and gamma sequences in the nucleus although these may not comprise the expressed TCR. Only one TCRβ CDR3 sequence will be expressed at the protein level. Figure created with BioRender.com.

The majority of human T cells are of the alpha/beta phenotype, expressing alpha and beta subunits (9, 10). The malignant T cells in MF are also predominantly alpha/beta T cells. As described above, alpha/beta T cells may have up to two rearranged TCRγ sequences in their nucleus. In addition, they can have either one rearranged TCRβ sequence (if the first rearrangement is functional), or up to two rearranged TCRβ CDR3 sequences in the nucleus. Only one TCRβ CDR3 sequence will be expressed in an alpha/beta T cell even though the cell may contain up to two rearranged gamma and two rearranged beta sequences in the nucleus (**Figure 1**).

### 2.2 Dominant T- cell clones in CTCL

**Figure 2:**
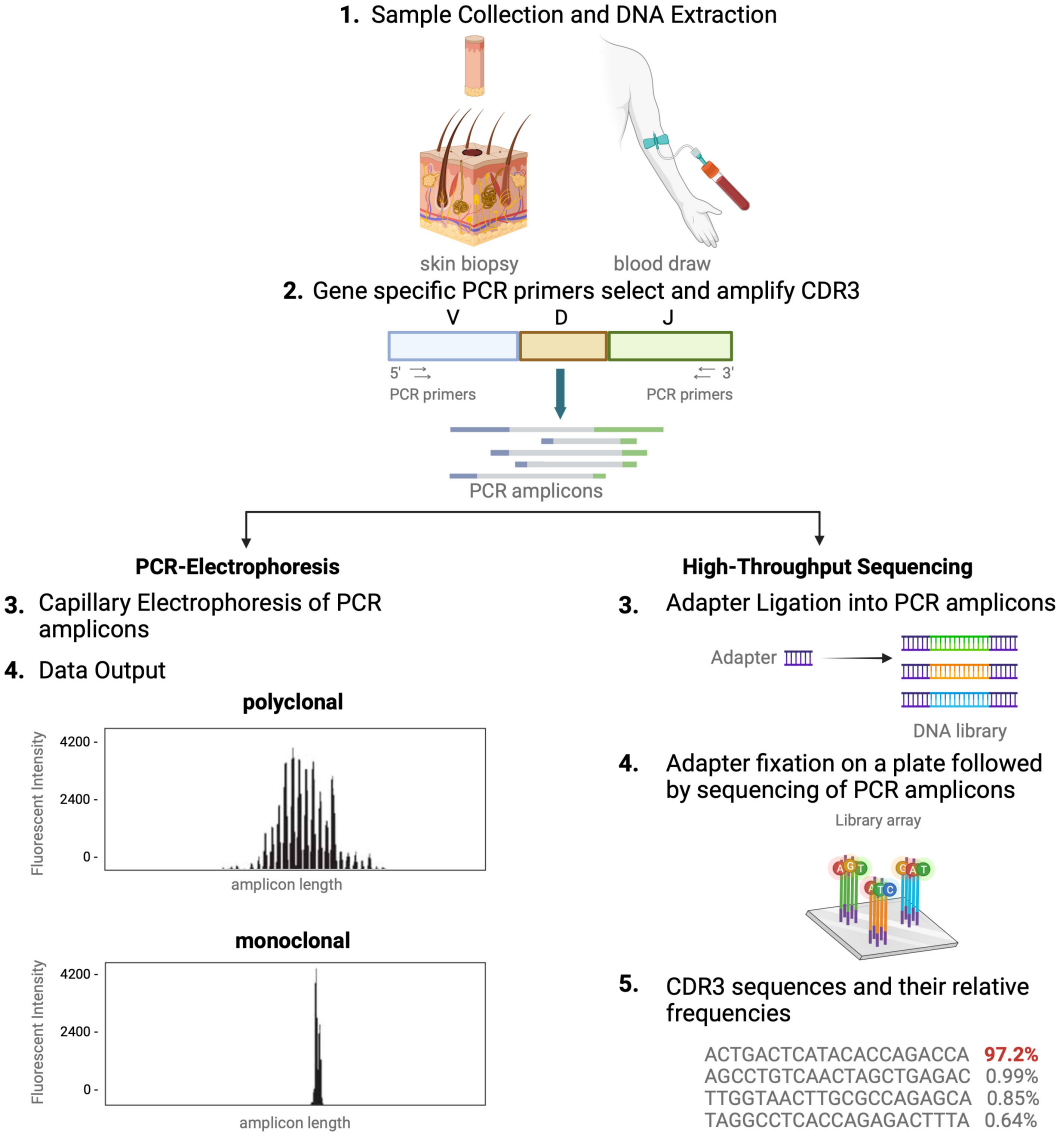
Comparison of TCR-sequencing methodology. A simplified stepwise illustration of the PCR-capillary electrophoresis and high-throughput sequencing method used to assess TCR gene rearrangements is depicted side-by-side. Sample collection from the patient is required to start from which DNA is isolated. This genomic material is subsequently subjected to amplification with PCR using primers designed to target V, D, and J sequences. The unique gene rearrangements are then analyzed with capillary electrophoresis (left side of figure), which generates a graphic presentation of monoclonal or polyclonal peaks whose length is used to distinguish individual TCRs from one another. High-throughput sequencing of T-cell receptors, immunosequencing, is shown in an abbreviated chronologic display (right side). Next generation sequencing (NGS) libraries for sequencing via adapter ligation (step 3, right), offered by services such as Illumina. The process of adapter ligation includes the complete set of sequencing primer binding sites required for single, paired end, and indexed reads. This eliminates the necessity for extra PCR procedures to incorporate the index tag and index primer sites. Then, NGS is used to produce the unique genetic sequence encoding each TCR and their relative frequencies. This figure was created with BioRender.com and adapted from “Next Generation Sequencing (Illumina)”, by BioRender.com (2023).

In healthy blood and skin, a large number of unique T cells, each marked by its distinct antigen binding specificity, contribute to a diverse T-cell repertoire. Conversely, in T-cell malignancies like MF, one T-cell clone tends to dominate. For this reason, detection of dominant T-cell clones has become a helpful and often necessary tool in the diagnostic evaluation and monitoring of CTCL (4, 7, 11, 12). Assessing T-cell clonality is particularly helpful when clinical, histopathologic, and immunophenotypic data do not fully support a CTCL diagnosis (7, 12). As detailed above, the unique signature of each T cell is determined by the TCR CDR3 sequences. Existing clonality assays interrogate TCR CDR3 sequences in the nucleus. There are two distinct methods of molecular analysis that can be used to assess T-cell clonality in CTCL. The first includes PCR paired with capillary electrophoresis (PCR-electrophoresis) (**Figure 2**), while the second method utilizes next generation sequencing (high-throughput sequencing) for detection of TCRγ and TCRβ gene rearrangements (**Figure 2**).

### 2.3 PCR-electrophoresis detection of T-cell clonality

Historically, TCR beta and gamma gene rearrangements in the skin of CTCL patients have been identified using PCR-electrophoresis (**Figure 2**). DNA is isolated from formalin-fixed, paraffin-embedded tissue blocks and amplified by PCR using BIOMED-2 primers targeting TCRβ and TCRγ V, D, and J sequences (10, 13). The DNA segments containing CDR3 gene rearrangements are amplified and separated based on size by electrophoresis. The standardized BIOMED-2 PCR protocol uses heteroduplex analysis or GeneScanning to detect clonal TCR gene rearrangements based on the length of the CDR3 region (10). In heteroduplex analysis of PCR products, monoclonal lymphoproliferations result in a single band via gel electrophoresis whereas polyclonal lymphoproliferations produce an indistinct band or smear. In GeneScanning, PCR primers are labeled with fluorochrome to detect PCR products. The fluorochrome-labeled products are separated by capillary electrophoresis where a normal, Gaussian distribution of multiple peaks represent polyclonality and a single peak implies monoclonality (**Figure 2**) (10). Generally, the intensity of the fluorescent peak suggests the frequency of the monoclonal TCR rearrangement in the sample. Overall, PCR-electrophoresis identifies CDR3 rearrangements using amplicon size as a proxy for T-cell clone identification but is unable to distinguish the exact sequences and frequencies of the CDR3s. Even though newer clonality assays like HTS-TCR can provide more precise information regarding the identity, frequency, and functionality of the TCR, the use of PCR-electrophoresis remains common in the workup of CTCL (**Tables 1**, **2**) (18).

**Table 1:** Supporting evidence for the clinical benefit of HTS-TCR in CTCL diagnosis and monitoring.

Clinical Use	References	Article Type	Evidence/Findings
Diagnosis	Kirsch et al., 2015 (4)	Retrospective cohort (n=39)	Sensitivity for T-cell clone detection - TCRγ PCR: 70%. - HTS-TCR: 100%
	Sufficool et al., 2015 (14)	Retrospective cohort (n=35)	Sensitivity for T-cell clone detection - TCRγ PCR: 44% - HTS-TCR: 85%
	Rea et al., 2018 (15)	Retrospective analysis (n=100)	- Specificity for T-cell clone detection - TCRγ PCR: 88% - HTS-TCR: 100%
Monitoring and Recurrence	Sufficool et al., 2015 (14)	Retrospective cohort (n=35)	- HTS-TCR is useful for tracking individual clones over time and detecting early MF recurrence
	de Masson et al., 2018 (16)	Retrospective analysis (n=309)	- Tumor clone frequency >25% is associated with worse outcomes and deems patients high risk
	Bhatti et al., 2023 (17)	Case Series (n=3)	- HTS-TCR aids in monitoring response to treatment and distinguishing MF progression from drug-related cutaneous adverse effects
Treatment	HTS-TCR use for guiding treatment has not yet been fully assessed and remains speculative		

TCRγ PCR, T-cell receptor polymerase chain reaction (otherwise referred to as PCR-electrophoresis); HTS, high throughput sequencing (otherwise referred to as next generation sequencing or immunosequencing).

**Table 2:** Summary of PCR vs. HTS-TCR data output.

	PCR	HTS-TCR
Clone identification	Deduced Determined by CDR3 base-pair length	Precise Determined by CDR3 genomic sequence
Frequency	Deduced Measured by magnitude of fluorescent dye attached to CDR3 PCR amplicon	Precise Measured by proportion of CDR3 PCR amplicon among other PCR amplicons
Clone tracking over time	Using CDR3 base-pair length	Using the identified CDR3 genomic sequence

### 2.4 T-cell clonality with immunosequencing

Immunosequencing is a PCR-based method that begins with amplification of rearranged TCR CDR3 sequences, similar to the PCR-electrophoresis method (**Figure 2**). The amplicons are then fixated on a plate and subjected to next generation sequencing. The data generated include a readout of receptor sequences and their relative frequencies at 100-fold greater sensitivity than PCR-electrophoresis (16). Overall, HTS-TCR provides the precise nucleotide sequence and frequency of all CDR3 segments in the sample. These data can be used to determine clonality based on criteria established and validated by the assay developer (19). Generally, to meet criteria for dominancy, the top frequency clone(s) need to be separated from other clones by some order of magnitude.

Several companies including Adaptive Biotechnologies, Illumina, CD Genomics, and Seqwell have developed publicly available HTS assays to detect human TCRs. The assays made available by Illumina, CD Genomics, and Seqwell are strictly for research use. ClonoSeq by Adaptive Biotechnologies is the only HTS assay that is FDA-approved for clinical use in hematologic malignancies (20). The ClonoSeq assay is utilized for immunosequencing of blood and skin in CTCL patients. ClonoSeq applies the following criteria to establish a dominant CDR3 sequence: the sequence must comprise at least 3% of all like sequences and at least 0.2% of the estimated total nucleated cells in the sample; the dominant sequence must be discontinuously distributed and be carried by at least 40 estimated genome equivalents in the analyzed sample (21).

ClonoSeq and platforms alike are unique from PCR-electrophoresis in their ability to identify and track the precise signature and frequency of the malignant T-cell clones. This facilitates high resolution malignancy detection and monitoring, enabling a personalized-medicine approach for management of CTCL. Of the HTS-TCR platforms available, ClonoSEQ has been further developed and FDA-approved (2018) to detect minimal residual disease (MRD) in bone marrow and peripheral blood of patients with other hematologic malignancies including acute lymphoblastic leukemia (ALL) and multiple myeloma (MM) (20). The power of this technology also has the potential to be harnessed in skin to evaluate T-cells in CTCL and to expand our understanding of the constituent immune repertoire. Analogous to its clinical use in other hematologic malignancies, we envision HTS-TCR as the future standard for identifying and monitoring T-cell clones in CTCL.

## 3 Utility of immunosequencing in skin

### 3.1 Diagnosis

Definitive diagnosis of CTCL, particularly in early stages, may take several years from initial manifestation of disease. This is partly due to the various benign dermatoses such as psoriasis and atopic dermatitis, among others, resembling MF in both clinical presentation and histologic characteristics (22). Nearly two decades ago, the International Society for Cutaneous Lymphoma (ISCL) developed a diagnostic algorithm aimed toward enhancing the identification of early-stage MF while also aiding in the differentiation from other non-malignant conditions (5). The algorithm assigns cumulative points using clinical, histopathological, immunohistochemical, and T-cell clonality criteria (5). If clinical and histologic findings are strongly suggestive of MF on their own, a diagnosis can be made. However, in cases where the diagnosis cannot be made based solely on these two parameters, there is a need for molecular and immunophenotypical analyses (5, 7). Overall, the presence of a dominant T-cell clone can be supportive of a CTCL diagnosis.

Immunosequencing has recently been introduced as a quantitative alternative to PCR-electrophoresis for the detection of T-cell clonality in the diagnostic evaluation of MF. The increased sensitivity and specificity associated with HTS-TCR allows for more accurate differentation of malignant clones in MF from benign clones seen in healthy individuals or inflammatory dermatoses (4, 18, 23). HTS-TCR is reported to have high sensitivity that ranges from 85-100%, which is improved from the sensitivity of PCR-electrophoresis ranging from 44-90% (**Table 1**) (4, 14, 18). This is clinically relevant because previous cases of MF that were reported to be clone-negative with PCR-electrophoresis were actually found to have a malignant clone when HTS-TCR was used (4). Furthermore, HTS-TCR identifies the precise nucleotide sequence of the T-cell clone with high specificity (15, 24). Although dominant clones can be identified and seemingly tracked with PCR-electrophoresis by base-pair length, this can occasionally be misinterpreted. For instance, clones of the same CDR3 length that were initially deemed identical through PCR-electrophoresis were later revealed to be distinct when their precise CDR3 DNA sequences were unveiled using HTS-TCR, although this is not always the case (15, 23).

The increased sensitivity and specificity of HTS-TCR is beneficial to establish the diagnosis of MF, especially in early-stage disease with nonclassical clinical presentation (**Table 2**). Immunosequencing can increase the likelihood of early diagnosis for MF patients with presentations mimicking other benign inflammatory diseases. In such instances, the primary benefit of early MF detection is providing opportunities to initiate appropriate MF treatment early in the disease course. This also prevents MF patients from receiving inappropriate treatments such as cyclosporine, TNF-alpha inhibitors, and dupilumab that may instigate MF progression (25–29). In addition, early-stage MF has been reported to progress in over 20% of patients within the first five years alone (30). Early MF detection and treatment may benefit this subset of high-risk early-stage patients who are likely to progress.

### 3.2 Prognostication

The prognosis for MF is variable, even among patients with the same stage of disease. In current clinical practice, two indices exist to aid in the prognostication of MF patients. These prognostic scales are known as the Cutaneous Lymphoma International Consortium (CLIC) score and the Cutaneous Lymphoma International Prognostic Index (CLIPi) (31, 32). Both scoring systems associate clinical characteristics with overall survival in early and late-stage MF (31). Emerging data suggest that HTS-TCR can provide an additional prognostic tool to distinguish individuals with MF who will experience rapid progression from those with an indolent clinical course. De Masson et al. introduced the concept of tumor clone frequency (TCF) which is defined as the percentage of the dominant TCRβ sequence among all rearranged TCRβ sequences (16). They showed that a TCF value greater than 25% in the skin of early-stage MF patients is associated with a significant reduction in overall survival (16). These findings indicate the clinical relevance of an abundant malignant T-cell clone in the skin at time of diagnosis and its effect on overall prognosis. As demonstrated with the application of TCF, immunosequencing has the potential to contribute to a more accurate prognostic model for MF.

Furthermore, analysis of HTS-TCR data can provide additional value for developing novel prognostic indicators. This has been shown in other solid and hematologic malignancies, where investigation of the immune repertoire has offered insight into underlying biological changes and may inform prognosis (33–35). Due to the complex nature of analyzing the immune repertoire comprised of both malignant and benign T-cells, there are limited peer-reviewed articles that address and define the T-cell repertoire in MF. However, a preliminary study suggests that changes in T-cell repertoire diversity may be related to the transformation event in large cell transformed MF and consequently contribute to its aggressive disease course (36). Considering the influence of the T-cell repertoire on disease progression in other malignancies, the utilization of HTS-TCR may further elucidate the role of the T-cell repertoire in MF. Nevertheless, additional comprehensive studies are needed to clarify the relationship between the T-cell repertoire and its impact on disease progression and prognosis in MF.

### 3.3 Monitoring disease course

Due to its ability to detect the precise genetic signature and frequency of malignant clones in CTCL, HTS-TCR can be employed for tracking malignant clones and disease monitoring over time (14, 19). Clone tracking is especially useful for evaluating treatment response at the molecular level, and for determining whether the development of new cutaneous lesions or rashes is due to disease progression, medication adverse effect, or alternative causes.

For CTCL, one of the key contributions of HTS-TCR lies in its capacity to quantify the presence of malignant clone(s) that persist following treatment. O’Malley et al. used HTS-TCR to quantify lesional disease burden with two distinct calculations: 1) tumor clone frequency (TCF) among T cells and 2) tumor clone frequency among all nucleated cells. Overall, they observed patients with lower residual frequencies of the malignant clones had better clinical responses than those with higher residual disease following treatment (37). This was not observed in patients who still harbored the malignant clone following treatment with either radiation or topical steroids (37). This is clinically significant as it demonstrates that even in patients who seemingly achieve clinical remission after treatment, the identification of persistent malignant clone(s) by HTS-TCR could predict prolonged disease trajectory or relapse. At the molecular level, Psoralen plus UVA (PUVA) was shown to reduce the malignant clone in low-burden disease but was unable to yield similar results in the skin of patients with high-burden disease (38). Similarly, topical Resiquimod gel, a Toll-like receptor 7/8 agonist, reduced the malignant T-cell clone in the majority of treated lesions and eradicated the malignant clone entirely in a subset of patients (39). In both studies regarding Resiquimod and PUVA, the correlation of malignant clone eradication with disease course was not described. Overall, while there is evidence that eradication of malignant clone indicates better response to treatment, the impact of therapy on skin malignant clone(s) and the associated disease course remains to be investigated further.

In patients with MF, it can be difficult to distinguish drug-related cutaneous adverse events from MF/SS progression in the skin. Some of the commonly used therapies for MF or Sézary Syndrome (SS) including mechlorethamine gel, a topical gel for treatment of early-stage patch/plaque MF, and mogamulizumab, an anti-CC chemokine receptor 4 monoclonal antibody for treatment of SS, are associated with cutaneous adverse events. Mogamulizumab associated rash (MAR) has been reported in up to 24-28% of patients treated with Mogamulizumab and is challenging to distinguish from MF disease progression (40, 41). In the pivotal MAVORIC trial, MAR grade 1–2 and 3 drug rashes occurred in 20% (36/184) and 4% (8/184) of mogamulizumab-treated patients, respectively (40, 41). Mechlorethamine gel 0.016% is associated with dermatitis in up to 56% of patients and can also be difficult to discern in patients with underlying MF (42, 43).

In MF patients treated with mogamulizumab or mechlorethamine gel, HTS-TCR is particularly useful for confirming the distinction between MF progression and cutaneous adverse events (44). In a recent study, immunosequencing was used in three MF patients to identify the malignant T-cell clones at the time of diagnosis and to track their frequencies over time. When the patients described in the study later developed MF-like rashes following treatment with mechlorethamine gel or mogamulizumab, HTS-TCR revealed lower or undetectable frequencies of the original malignant clones in the biopsies of the newly involved areas of skin. These findings, in addition to other histopathologic features, confirmed the diagnosis of MAR or mechlorethamine gel associated hypersensitivity dermatitis (44).

Similarly, HTS-TCR can be used to distinguish between two co-existing malignancies in one patient. The emergence of a new lymphoma in a patient with a pre-existing malignancy is a known phenomenon in the setting of immunosuppression. Several cases have reported composite lymphomas, a combination of two different B-cell or T-cell lymphomas, or concomitant B-cell and T-cell lymphomas in this population (45, 46). It is also possible for primary lymphomas to metastasize many years after initial diagnosis (47). Therefore, in patients who develop an additional malignancy, it is critical to determine whether the co-existing malignancies derive from the same origin or if they are genetically distinct (48). In most reported cases, PCR-electrophoresis assays were used to aid in the differentiation between the concurrent processes. However, due to the superior specificity and accuracy of HTS-TCR for malignant clone identification, its application may be better suited to distinguish the unique clonal origins of co-existing malignancies.

Beyond the clinical application of HTS-TCR, immunosequencing can be used to meticulously track clonal evolution, or how the unique malignant T-cell clones change over time. This has been exemplified by the discovery of intraclonal CDR3 variants, defined by one single base substitution in the constituent dominant TCRβ CDR3 sequences. The novel detection of intraclonal CDR3 variants was associated with changes in clinical and histopathologic behavior, including progression to tumor-stage MF, new-onset large cell transformation, and resistance to treatment (49). This observation demonstrates the importance of interrogating the CDR3 sequences throughout the course of disease. The idea of subclonal T-cell evolution in MF has been appreciated in other genomic studies, but preliminary studies have not been able to identify a common series of genomic events in early-stage disease (**Table 3**) (50). Future HTS-TCR studies are needed to further delineate the natural evolution of MF.

**Table 3:** Utility of HTS-TCR in skin: key points.

T-cell clonality tests are now an essential component of the diagnostic toolkit for CTCL
Immunosequencing precisely identifies and quantifies the genetic sequence of TCR CDR3 of malignant clones
Increased sensitivity and specificity of HTS-TCR compared to PCR aids in diagnosis of early-stage MF
HTS-TCR can provide TCF, a measure that has prognostic value
HTS-TCR can be used to monitor the treatment response by tracking malignant T-cell clones in the skin and blood after treatment
HTS-TCR can help distinguish between disease recurrence, treatment related cutaneous adverse events, and co-existing malignancies

## 4 Utility of immunosequencing in blood

### 4.1 Blood staging and diagnosis

Although MF is more commonly confined to the skin, it can progress to involve blood, lymph nodes, and solid organs. Detection of blood involvement in MF is an important and necessary diagnostic step which must be completed at initial staging and repeated over time based on clinical indications. Detection of blood involvement originally relied on cytology with observation of Sézary cells, marked by their cerebriform nuclei (51). If more than 1000 Sézary cells/µL of blood are observed, blood involvement is confirmed (7). More recently, MF diagnosis and characterization of blood involvement has been advanced with the incorporation of immunophenoytoping by flow cytometry. MF and SS cells in blood are often evaluated by identifying expression of CD2, CD3, CD4, CD8, CD5, CD7, CD26, CCR4, TCRβ, and CD45RO on cell surfaces (52). Specifically, the standard for assessing blood involvement in most available flow cytometry assays is detection of CD4+/CD26- cells or CD4+/CD7- among T cells and lymphocytes (7).

Blood involvement or B staging in MF is classified as B_0_, B_1_, or B_2_ by quantification of aberrant lymphocytes in blood. B_2_ staging signifies frank blood involvement by MF cells, or Sézary Syndrome. MF patients classified as B_2_ by flow cytometry have a less favorable prognosis compared to B_0_ or B_1_ patients (53). According to most recent guidelines proposed by the NCCN and United States Cutaneous Lymphoma Consortium (USCLC), the absolute number of CD4+/CD26- cells or CD4+/CD7- cells determines B classification (7, 12). B_0_ is defined by <250/µL of CD4+/CD26- cells or CD4+/CD7- cells, while B_2_ is marked by > 1000/µL of CD4+/CD26- cells or CD4+/CD7- cells as identified by flow cytometry. B_1_ is described by failure to meet criteria for either B_0_ or B_2_ (7, 12).

### 4.2 T-cell clonality in blood and its relevance to prognosis

While flow cytometry is predominantly used for determination of blood staging in MF, T-cell clonality assays of blood are also used to evaluate blood involvement. According to NCCN/USCLC guidelines, if an identical T-cell clone in the skin and blood is detected, patients can more specifically be classified as B_1B_/B_2B_ in contrast to B_1A/2A_ where no blood clones are detected (7, 12). Not surprisingly, MF patients with B_2_ status are more likely to have a dominant T-cell clone in the blood that matches the clone in the skin as identified by clonality assays (7, 11, 12). Additionally, detection of a dominant T-cell clone in the blood of MF patients regardless of blood staging has been shown to portend a poor prognosis (32, 54).

Blood clonality assays can be performed by methods such as TCR-Vß flow cytometry, PCR-electrophoresis, and HTS-TCR. Flow cytometry of TCR-Vß is limited by available antibody panels, which cover only 70% of all TCR-Vß chain variables, leading to potential omission of diagnosis or detection in a subset of patients (55, 56). Use of PCR-electrophoresis for detection of clonal TCR gene rearrangements in blood has reportedly high false negative rates up to 25% and can be unreliable in the evaluation of early-stage MF (16, 22). However, like its use in skin, immunosequencing can evaluate T-cell clonality in the blood with a sensitivity 100 times greater than that of flow cytometry and PCR-electrophoresis (57). HTS-TCR allows for identification of the exact CDR3 sequence and its absolute frequency (7, 57). As a result, HTS-TCR enables a more precise means to determine whether detected malignant clones in the skin match those in the blood (7, 11, 12).

Several studies have explored the impact of blood T-cell clonality on prognosis (54, 58–63). These studies have shown that the presence of a peripheral blood T-cell clone is linked to a less favorable prognosis, worse treatment response, and a shorter time to initiation of systemic treatment (54, 58–63). However, the presence of a dominant peripheral blood T-cell clone alone may not be sufficient to predict prognosis and needs to be considered in the context of dominant skin T-cell clones. Several groups began to interrogate the relationship between dominant blood and skin clones and their impact on prognosis using either PCR-electrophoresis, southern-blotting, or HTS-TCR assays. These studies show that when dominant T-cell clones in the blood match those in the skin, patients have a higher risk of disease progression and poor prognosis (**Table 4**) (54, 58, 60, 64). On the other hand, when dominant clones are present in both blood and skin but distinct from one another, prognosis is more favorable (58, 60). Since HTS-TCR can identify the exact CDR3 sequences, it offers a more accurate means to establish whether dominant clones in blood and skin are truly identical. Of the studies that evaluated the relationship between dominant blood and skin clones, our group was the first to utilize HTS-TCR in a large cohort of early-stage MF patients. We found that patients with different, or ‘discordant’, dominant T-cell clones in blood and skin or no dominant blood clones had a longer time to systemic treatment compared to those with identical clones in blood and skin (58).

**Table 4:** Utility of HTS-TCR in blood: key points.

HTS-TCR is more sensitive and specific than flow cytometry or PCR for detection of clonal T-cells in blood.
HTS-TCR can establish clonal associations between dominant T-cell clones in the skin and blood.
Detection of identical dominant T-cell clones in both the skin and blood portends a worse prognosis.
Detection of a polyclonal or discordant dominant blood T-cell clones portends a more favorable prognosis.
HTS-TCR is being used in other cancer indications to track MRD and has potential for future use in CTCL.

Overall, there is evidence that clonal associations between dominant T-cell clones in the skin and blood of MF need to be evaluated. Since with PCR-electrophoresis, identification of T-cell clones is inferred by base-pair length (**Table 2**), this assay cannot confirm with high accuracy that clones are identical in blood and skin (10). HTS-TCR presents a more precise assay to compare the identity of dominant clones in the skin and blood (**Table 4**). Larger prospective multi-center studies are needed to evaluate the prognostic relevance of blood and skin T-cell clonal associations.

### 4.3 Disease monitoring

Tracking immunophenotypes based on absolute counts of CD4^+^/CD26^-^ cells or CD4^+^/CD7^-^ cells identified by flow cytometry continues to be the standard for monitoring blood involvement in CTCL. Although the application of HTS-TCR in blood for CTCL monitoring continues to be evaluated, there are distinct advantages to its implementation in clinical practice that warrant discussion. One potential application of HTS-TCR is to monitor low-level blood or skin tumor burden after treatment, also known as detection of minimal residual disease (MRD). In other hematologic malignancies such as chronic lymphocytic leukemia (CLL) and acute T lymphoblastic leukemia, MRD is used for recognizing early disease recurrence following allogeneic stem cell transplant (23, 65, 66). Although MRD detection is not currently a component of CTCL management, Weng et al. demonstrated that HTS-TCR reliably identifies 1 tumor cell within 50,000 peripheral blood cells in CTCL patients following allogeneic stem cell transplant (23). As a result of its high specificity and sensitivity, HTS-TCR has the potential to detect MRD in CTCL patients (**Table 4**). The application of immunosequencing for MRD monitoring in CTCL needs to be studied in larger, prospective studies. However, if it is adopted in the future of CTCL clinical practice, use of HTS-TCR for MRD tracking may aid in revealing early disease relapse, allowing for shortened time to intervention, and theoretically improved outcomes. It is likely that MRD positive patients may exhibit worse outcomes and require more aggressive treatment compared to MRD negative patients.

## 5 Advancing CTCL research with HTS-TCR

The growing utilization of immunosequencing for CTCL not only serves to augment clinical practice but also to enhance our understanding of malignant and non-malignant T cells in skin and blood and their changes over time. By providing the precise genetic sequences and absolute abundance of CDR3s, HTS-TCR offers a high-resolution view of the immune repertoire. Specifically, HTS-TCR data can be used to determine T-cell repertoire diversities in blood and skin (36). In addition, HTS can assess the T-cell repertoire overlap between blood and skin or various skin lesions over time, as demonstrated by Joffe et al. (58). As discussed above in section 3.3, the utility of HTS-TCR extends to identifying mutations that can arise in the dominant CDR3s. The existing study detected mutated versions of the malignant CDR3 clones that became dominant themselves (49). It would be interesting to further explore the prevalence and clinical relevance of mutated CDR3 clones that do not reach the level of dominance. Finally, HTS-TCR now provides a means to study MRD in CTCL patients, heralding a new era of inquiry into disease monitoring and management (23).

## 6 Challenges and limitations of HTS-TCR in CTCL

Although HTS-TCR has significant clinical value, it has introduced us to novel challenges throughout our six-year experience of HTS-TCR utilization at Thomas Jefferson University. The clinical-grade HTS-TCR test used in our center (Adaptive Biotechnologies) identifies both dominant TCRβ and TCRγ sequences. Receiving two separate clonality results for TCRβ and TCRγ can be confusing to clinicians. These results become especially challenging when TCRγ identifies a dominant clone and TCRβ does not, or vice versa. In such cases, clinicians may falsely attribute having a dominant TCRγ clone and no dominant TCRβ clone to a gamma/delta T-cell lymphoma. However, as described in section 2.1, having dominant TCRγ sequences in the nucleus does not signify that the cell is expressing the gamma sequence and is of gamma/delta lineage. Rather, there can be up to two rearranged TCRβ and TCRγ alleles in the nucleus of one alpha/beta T cell (**Figure 1**), though even with HTS-TCR these cannot be discerned. Additionally, the number of dominant clones detected by TCRγ versus TCRβ assays generally do not align. For example, there may be three dominant TCRγ and two dominant TCRβ clones each with a different frequency in one skin biopsy. While the differences between TCRγ and TCRβ immunosequencing results may be due to the unique sensitivities of each assay, when multiple clones are detected, additional challenges arise.

It is not uncommon for multiple dominant sequences to be identified within CTCL skin biopsy specimens, and the clinical significance of detecting multiple clones is not yet clear (49). This is particularly challenging to interpret when three or more dominant sequences are detected. Given that up to two TCRβ and TCRγ rearrangements can exist in the nucleus of one T cell, when three or more dominant sequences are identified with HTS-TCR, there is a high likelihood of there being more than one malignant T-cell clone. In cases where multiple dominant clones are detected, a few variations of results are possible. For example, multiple dominant clones may each be malignant, only one clone may be malignant while the others are not, or the dominant clones detected may be non-malignant. The range of possibilities with detection of more than one clone can further complicate clinical interpretation. It is possible that future development of clinical-grade RNA-based sequencing assays will allow for a better understanding of the dominant sequences most relevant to the patient’s disease.

An additional challenge presented by HTS-TCR includes the identification of mutations in dominant T-cell clones. As previously mentioned in section 3.3, intraclonal CDR3 variants have been detected and described at our institution. The emergence of these mutated clones were associated with progression of disease as well as resistance to treatment (49). However, the clinical significance of intraclonal CDR3 variants needs to be studied further. In addition, discrepancies in the number of dominant clones detected by HTS-TCRγ versus HTS-TCRβ can contribute to challenges in clinical interpretation. These discrepancies can be due intratumoral clonal heterogeneity as demonstrated elegantly by the Gniadecki group (67, 68).

Another barrier to widespread implementation of immunosequencing is the associated overhead cost, which is actively changing as the platform gains popularity. In addition, cost-coverage by insurance companies is an ongoing issue, as most insurance carriers refuse to reimburse immunosequencing costs. In the absence of insurance, the out-of-pocket cost for each ClonoSeq test ranges from $1,500.00 to $2,500.00.

Like PCR, the sensitivity of HTS-TCR is limited by the quantity or quality of tissue, or the amount of DNA that can be probed. Specifically, section thickness and cell size from samples may impact amplification and representation of gene segments (24, 57). Furthermore, HTS-TCR does not distinguish between α/β or γ/δ chains of a specific TCR or whether a T-cell clone is CD4+ or CD8+. This represents a major limitation, as not being able to distinguish between different phenotypes of T cells or the presence of reactive CD8+ clones in skin or blood can hinder diagnosis and management of CTCL patients. Lastly, some early-stage MF patients will not demonstrate clonality, despite clinical and histological data supporting a diagnosis of MF (60). Therefore, clinicians cannot always rely on HTS-TCR to make or exclude a diagnosis of CTCL and must consider HTS-TCR results in context of clinical and histopathologic findings. A summary of the challenges and limitations are described in **Table 5**.

**Table 5:** Challenges and limitations of HTS in clinical practice: key points.

Multiple dominant T-cell clones may be detected with unclear clinical significance.
Intraclonal CDR3 variants pose a challenge to clinicians because of ambiguous clinical significance.
Cost and lack of insurance coverage may be prohibitive to widespread adoption of HTS-TCR.
Quantity of tissue or amount of DNA probed may impact representation of gene segments.
Unable to distinguish between α/β or γ/δ chains of a specific TCR or whether a T-cell clone is CD4+ or CD8+
May not detect dominant clone in early-stage MF, making other factors like clinical presentation and histology important

## 7 Conclusion

Immunosequencing, also known as HTS-TCR, offers several unique advantages for the evaluation and management of CTCL. While healthy patients have a diverse population of T-cells, each with distinct antigen binding specificity afforded by their TCRs, CTCL patients often have aberrant proliferations of dominant T-cell clones. Per NCCN guidelines, clonality assays are now requisite for the diagnostic evaluation of the most common CTCL subtype, Mycosis Fungoides (7). HTS-TCR is a newer diagnostic technique that allows for improved detection and quantification of dominant T-cell clones in the skin and blood. It can be used to prognosticate patients based on the frequency of their dominant T-cell clones and to monitor persistence or eradication of dominant clones more accurately over time. HTS-TCR is also clinically beneficial for differentiating disease progression from cutaneous adverse reactions or other benign inflammatory dermatoses. Immunosequencing can correlate the clonalities of the skin and blood with one another for individual patients. In addition to the clinical value HTS-TCR provides, it has broader applications in research, enabling further investigation of blood and skin T-cell repertoires and clonality in CTCL.

## Author contributions

JM: Writing – original draft, Writing – review & editing. LG: Writing – original draft, Writing – review & editing. DJ: Writing – original draft, Writing – review & editing. SB: Writing – review & editing. NN: Conceptualization, Data curation, Funding acquisition, Investigation, Methodology, Project administration, Resources, Supervision, Visualization, Writing – original draft, Writing – review & editing.
